# Retinal manifestations in adult T-cell leukemia/lymphoma virus type 1 (HTLV-1)

**DOI:** 10.1186/1742-4690-12-S1-P74

**Published:** 2015-08-28

**Authors:** Harold Merle, Jean-Côme Meniane, Christophe Deligny, Yves Plumelle, Angélique Donnio, Samy Chraibi, Albert Jean-Charles

**Affiliations:** 1 Department of Ophthalmology, Centre Hospitalier Universitaire de Fort-de-France, Martinique (French West Indies; 2 Department of Hematology, Centre Hospitalier Universitaire de Fort-de-France, Martinique (French West Indies; 3 Department of Medicine, Centre Hospitalier Universitaire de Fort-de-France, Martinique (French West Indies

## 

To describe retinal manifestation throughout the course of adult T-cell leukemia (ATL) linked to infection by the human T-cell lymphotropic virus type-1 (HTLV-1). Retrospective case series of patients showing complicated ATL intraocular manifestation. Complete eye exam was performed, with among other test fluorescein angiography, indocyanine green angiography, and optical coherence tomography. Sample of the vitreous was collected for observation. Three patients showed intraocular complications linked to ATL, among the 175 ATL cases diagnosed in Martinique between 1983 and 2013. They were bilateral under retinal infiltrates associated with intermediate uveitis. In 2 cases, the ATL diagnosis was known. The retinal infiltrates visible through angiography allowed to make the ATL diagnosis for the third case. ATL type cells presence was confirmed in the vitreous for one patient. Despite chemotherapy, infiltrates progress in a centripetal pattern to reach the posterior pole. They were associated with vasculitis, thus reducing visual acuity to simple luminous perception. Retinal manifestation throughout ATL is very rare. From the onset of the disease, the presence of characteristic under retinal infiltrates allow right away the diagnosis of ATL for an HTLV-1 virus seropositive.

